# Supplementary material for: Covid-19 by Race and Ethnicity: A National Cohort Study of 6 Million United States Veterans
Source: medRxiv. 2020 May 17:2020.05.12.20099135. Preprint. [Version 1] doi: 10.1101/2020.05.12.20099135 (PMC7273292; doi:10.1101/2020.05.12.20099135)
Supplement: 1 [file NIHPP2020.05.12.20099135-supplement-1.pdf]

medRxiv preprint doi: https://doi.org/10.1101/2020.05.12.20099135.this version posted May 18, 2020. The copyright holder for this preprint (which was not certified by peer review) is the author/funder, who has granted medRxiv a license to display the preprint in perpetuity.
It is made available under a CC-BY 4.0 International license .

Appendix

Table S1. International Classification of Diseases, Tenth Revision, Clinical Modification (ICD-10-CM) Diagnosis Codes

	ICD-10-CM codes
**Comorbid conditions**	
**Asthma**	J45.X
**Cancer**	
Cancer	C00.X-C43.X, C45.X-C76.X, C80.X-C96.X, C7A.X
Metastatic cancer	C77.X-C79.X
**Chronic obstructive pulmonary disease**	J41.X, J42.X, J43.X, J44.X
**Chronic kidney disease**	I12.0X, I13.1X, N03.2X-N03.7X, N18.X, N19.X, N05.2X-N05.7X, N25.0X, Z49.0X - Z49.2X, Z94.0X, Z99.2X
**Diabetes mellitus**	E08.X, E10.X, E11.X, E13.X
**Hypertension**	I10.X-I13.X, I15.X, I16.X
**Liver disease**	
Hepatitis B virus	B16.X, B18.0X, B18.1X, B19.1X, Z22.51
Hepatitis C virus	B17.10, B17.11, B18.2, B19.20, B19.21, Z22.52
Hepatic decompensation	I85.01, K65.2, K70.31, K72.1X, K72.9X, K76.7, R18.8
Other mild liver disease	B18.8X, B18.9X, K70.0X-K70.2X, K70.30, K70.9X, K71.3X-K71.5X, K71.7X, K73.X, K74.X, K76.0X, K76.2X-K76.4X, K76.8X, K76.9X, Z94.4
Other severe liver disease	K76.6, I85.00, I85.9X, I86.4, I98.2X, K70.4X, K71.1X, K76.5X
**Vascular disease**	
Acute myocardial infarction	I21.X (not including I21.AX), I22.X
Cardiomyopathy	I42.X, I43.X
Coronary heart disease	I20.X, I24.X, I25.10, I25.110, I25.2, I25.3, I25.41, I25.42, I25.5, I25.700, I25.710, I25.720, I25.730, I25.750, I25.760, I25.790, I25.8X, I25.9
Heart failure	I09.9, I11.0, I25.5, I13.0, I13.2, I50.X, P29.0
Cerebrovascular accident	I60.X-I69.X, G45.X, G46.X, H34.0
Peripheral vascular disease	I70.X, I71.X, I73.1-I73.9, I77.1, I79.0, I79.2, K55.1X, K55.8X, K55.9X, Z95.8X, Z95.9
**Substance use**	
**Alcohol use disorder**	F10.1X, F10.2X

medRxiv preprint doi: https://doi.org/10.1101/2020.05.12.20099135.this version posted May 18, 2020. The copyright holder for this preprint (which was not certified by peer review) is the author/funder, who has granted medRxiv a license to display the preprint in perpetuity.
It is made available under a CC-BY 4.0 International license .

Table S2. Associations with testing positive for Covid-19 over time

	**Positive test result among tested**	
	**February 8 to April 3**	**April 4 to May 4**
Number complete cases	14,813	38,093
Number of events	2,391	2,695
Race/ethnicity		
White	ref	ref
Black	2.59 (2.29-2.94)	1.64 (1.48-1.82)
Hispanic	1.77 (1.46-2.14)	1.71 (1.45-2.00)
Age, years		
20-39	0.80 (0.65-0.97)	0.94 (0.79-1.12)
40-49	0.79 (0.65-0.96)	1.10 (0.92-1.31)
50-59	0.87 (0.74-1.02)	1.10 (0.96-1.25)
60-69	ref	ref
70-79	1.10 (0.95-1.29)	1.11 (0.98-1.26)
≥80	1.02 (0.82-1.26)	1.26 (1.08-1.47)
Sex, male vs. female	2.31 (1.92-2.78)	1.42 (1.22-1.66)
Residence type, urban vs. rural	1.26 (1.05-1.52)	1.41 (1.22-1.64)

Abbreviations: Covid-19, coronavirus disease 2019; OR, odds ratio; CI, confidence interval

Notes: OR (95% CI) from conditional logistic regression modeling stations with five or more Covid-19 cases and conditioning on VA site of care. Additionally adjusted for baseline comorbidity (asthma, cancer, chronic kidney disease, chronic obstructive pulmonary disease, diabetes mellitus, hypertension, liver disease, vascular disease), substance use (alcohol consumption, alcohol use disorder, smoking status), and medication history (angiotensin converting enzyme inhibitor, angiotensin II receptor blocker)

[a]Low number of mortality events in age groups 20-39 (0 events) and 40-49 (3 events) thus grouped with 50-59 (23 deaths)
